# Is the threatened land crab *Cardisoma guanhumi* conquering human‐dominated systems?

**DOI:** 10.1002/ece3.10737

**Published:** 2024-04-25

**Authors:** José M. Riascos, Levy D. Obonaga, Jhostin Ramos

**Affiliations:** ^1^ Corporación Académica Ambiental Universidad de Antioquia‐Sede Ciencias del Mar Turbo Antioquia Colombia; ^2^ Corporation Center of Excellence in Marine Sciences – CEMarin Bogotá Colombia; ^3^ Programa de Doctorado en Ciencias del Mar Universidad de Antioquia Turbo Colombia; ^4^ Programa de Ecología de Zonas Costeras Universidad de Antioquia‐Sede Ciencias del Mar Turbo Antioquia Colombia

**Keywords:** blue land crabs, croplands, geographical range extension, habitat destruction, threatened species, urbanization

## Abstract

Land use changes are heralded as a major driver of biodiversity loss. However, recent findings show that cities, perhaps the most radical habitat transformation, sustain increasing numbers of threatened species. This emerging trend has been mostly chronicled for vertebrates from landlocked cities, although loss of biodiversity and rates or urbanization are higher in coastal marine systems. To advance our understanding on how threatened species may conquer human‐dominated systems, we studied the threatened edible crab *Cardisoma guanhumi* and assessed how it is proliferating in croplands and urban systems at different spatial scales and whether populations show consequences of long‐term exploitation. We gathered the data on crab populations covering the whole distribution range, including three countries reporting this as a threatened species. The abundance, distribution, and size structure of crab populations among different land uses at local scales were compared and published data for populations thriving in different habitats throughout their distribution range were compiled. We found that at local scale this species is able to thrive in natural and human‐disturbed habitats, where food sources are heavily altered. At larger scales, the species showed no differences in abundance and size structure among natural and anthropogenic habitats. In areas near the southern distribution edge, crab populations were more abundant and composed of larger animals in urban areas and croplands than those in natural habitats, suggesting that human‐disturbed systems are stepping stones to extend the geographic range. However, we found a long‐term reduction in maximum body size, exacerbated by land use changes, that likely reflects exploitation regimes consistently targeting larger crabs. Despite its status as a threatened species, the long history of human exploitation combined with livestock farming practices may explain the proliferation of this crab in human‐dominated systems, which emphasize the need to consider conservation in human‐dominated systems.

## Graphical abstract

Land use change has been recognized as a major contributor to habitat destruction, the main cause of biodiversity loss. However, recent findings show that cities – probably the most radical habitat transformation – may sustain threatened species. This intriguing trend has been mostly chronicled in small‐scale studies of vertebrate communities from landlocked urban systems, despite the fact that loss of biodiversity and rates or urbanization are greatest in coastal systems. To advance our understanding on the generality and drivers of this development, we studied the threatened edible crab *Cardisoma guanhumi* and asked whether crab populations are thriving in human‐altered areas across its biogeographic range and whether populations are showing responses to long‐term exploitation. Despite its status as endangered species it is proliferating in human‐disturbed habitats, where food regimes are heavily altered. Indeed, human‐disturbed habitats may act as stepping stones to extend its geographic range. Thus, our work adds evidence that the future of many endangered species will be defined beyond the boundaries of parks and reserves. It is a call to overcome a long‐standing tradition in conservation biology of focusing on wild species in “natural” ecosystems and to address the need of conservation in expanding human‐dominated systems.

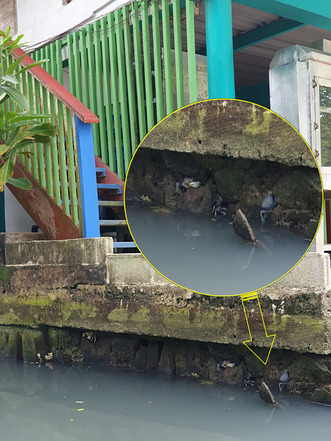

## 1 INTRODUCTION

The ongoing urban expansion has been widely depicted as a process intrinsically deleterious for biodiversity. Urbanization alters all the basal abiotic and biotic properties that govern ecosystems, including disturbance regimes, soil properties, nutrient loads, pollution levels, arrival and competition from non‐native species, herbivory and predation rates (Alberti, 2008; Pickett & Cadenasso, 2009). In fact, the conversion of wild regions into urban areas and other systems produced or managed by humans has been described as a process of habitat destruction (i.e. the alteration of a wild habitat to a degree that it no longer supports the species it originally sustained; Laurance, 2010) and a major cause of biodiversity loss (e.g., Laurance, 2010; Pimm & Raven, 2000). Moreover, unsustainable harvesting typically observed near large urban areas (Di Minin et al., 2019) often leads to local extirpations or long‐lasting population changes (Allendorf & Hard, 2009; Brans et al., 2017), further increasing biodiversity loss.

Current approaches in biological conservation assumes a causal link between habitat change and human exploitation and biodiversity loss (e.g., Krauss et al., 2010; Tilman et al., 2001), which suggest that the bulk of species highly adapted to a given habitat are passively disappearing while their natural habitats are being destroyed. However, this narrative is being challenged by recent findings showing that cities and similar human‐dominated systems may harbor high numbers of threatened species; in some instances, more than in equivalent wild areas (Ives et al., 2016). Indeed, urban areas with a long history of intense human exploitation may have a higher potential for the recovery of threatened species, as observed for the smalltooth sawfish *Pristis pectinata*, now proliferating in urbanized coastal waters off Miami (McDonnell et al., 2020). A diverse set of threatened species are conquering human‐dominated systems, including plants (e.g. Planchuelo et al., 2019), birds (e.g, Geary et al., 2021; Ives et al., 2016) and mammals (Busschots et al., 2020; Maclagan et al., 2018; Van Helden et al., 2020). Indeed, the size of urban populations of threatened species may be larger than their rural counterparts (Pagel et al., 2018). Thus, we must reconsider the traditional approach of avoiding biodiversity loss through the protection of wild, untouched habitats and assess both the mechanisms and the generality of the colonization of human‐dominated systems by threatened species.

To date, unfortunately, the bulk of knowledge on the urbanization of threatened species comes from the study of vertebrates in mainland areas, with less attention to the more speciose groups in coastal areas. This is so despite the fact that (i) human‐related compositional changes and losses of biodiversity are more pronounced in tropical marine than in terrestrial systems (Blowes et al., 2019) and (ii) coastal tropical areas will see some of the greatest urbanization rates over the next century (Branoff, 2017).

On the other hand, scale represents a challenge in our quest to understand how species respond to urbanization. For instance, it is common that regions defined as ‘urban’ in a given study contain in fact a heterogeneous composition of land covers that include small urban and rural patches, thus obscuring the assessment of drivers of the urbanization of endangered species (Ives et al., 2016). This also reflects a more general issue: the lack of adequate definitions of urbanness that would allow the objective quantification of urban gradients (Branoff, 2017).

To better understand why populations of threatened species are inhabiting urban areas we studied the blue land crab *Cardisoma guanhumi* (Latreille, 1828), a circumtropical species distributed from Florida, USA, to São Paulo, Brazil (Rathbun, 1918). This crab is an important resource in small‐scale fisheries in Latin America and is currently included in lists of IUCN threatened species in Brazil (Critically endangered; SIBBr, 2021), Venezuela (Vulnerable; Carmona‐Suárez, 2015) and Colombia (Vulnerable; Ardila et al., 2002), where the threats are assumed to be the loss and fragmentation of habitats and overexploitation. As harvesting may be triggering population‐level responses (Allendorf & Hard, 2009; Brans et al., 2017) that interact with other human‐related disturbances, these dimensions must be studied together. Therefore, we addressed the following questions: (1) whether populations of *C. guanhumi* are developing in human‐disturbed systems through its geographical range and (2) whether populations are showing consequences of long‐term exploitation. To achieve this, we first compared the abundance, size structure and spatial distribution patterns of populations simultaneously occurring in unvegetated urban areas, croplands and mangrove forest patches at the local scale – a small bay at the southern Caribbean coast of Colombia. Thereafter, based on a comprehensive compilation of data from the literature we compared the abundance and size structure of crab populations occurring in natural and human‐dominated systems through the Americas and assessed if cumulative effects of fishery and habitat alteration during the last 60 years translate into a decrease in mean abundance and body size of this crab.

## 2 MATERIALS AND METHODS

### 2.1 Comparisons of populations of *Cardisoma guanhumi* among land covers in Sapzurro bay

#### 2.1.1 Study area

**FIGURE 1:**
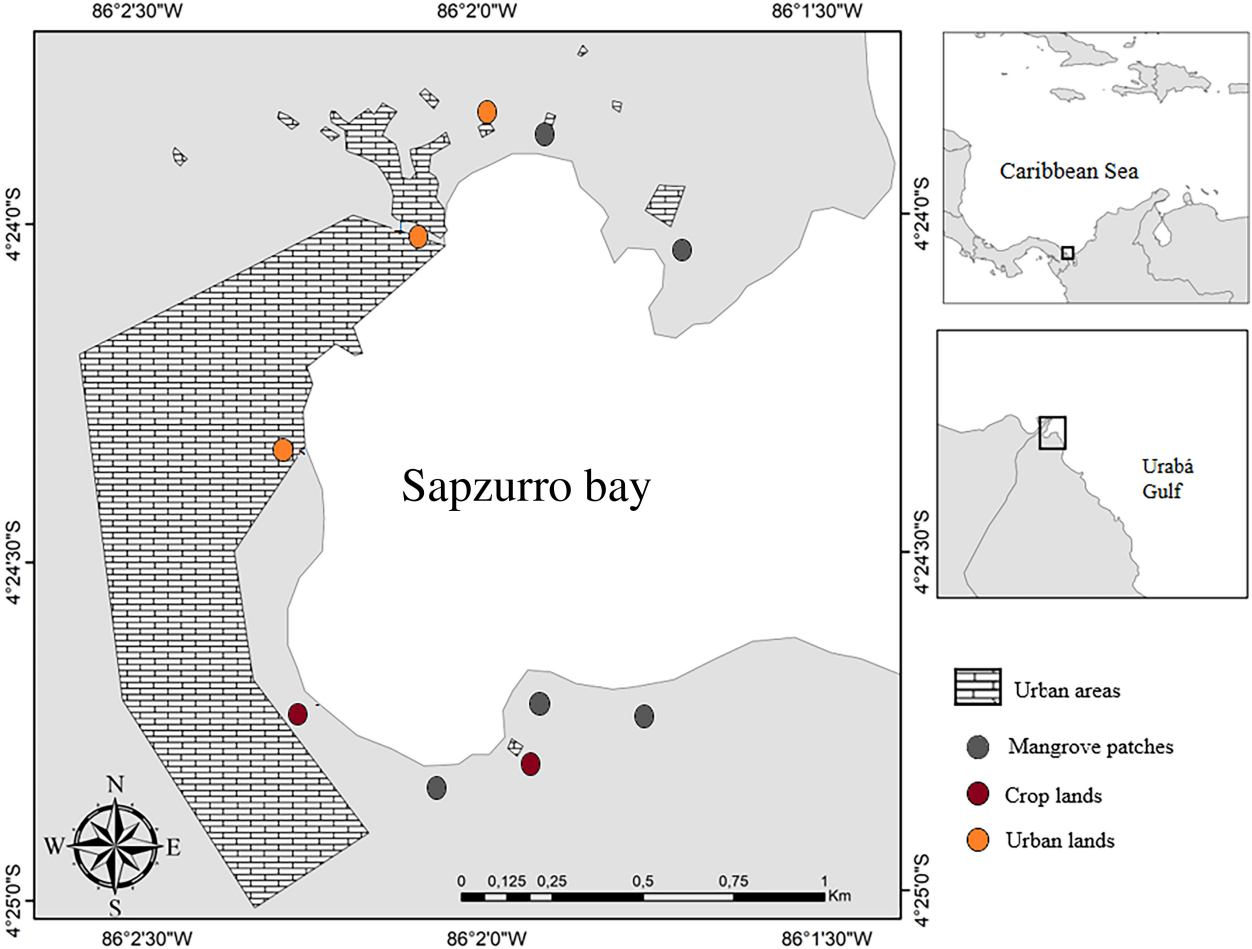
Map of Sapzurro bay, Colombian Caribbean. The map shows the ten places with contrasting land uses where *Cardisoma guanhumi* was sampled.

Located near the Colombia‐Panama border, Sapzurro is an east‐facing shallow embayment located in the western side of the Urabá Gulf. The Gulf is part of the Chocó‐Darien Global Ecoregion, a globally recognized biodiversity hotspots prioritized for conservation due to the high levels of biodiversity and endemism (Fagua & Ramsey, 2019). Despite this, the coalescence of outrages and conflicts that characterized the aftermath of European invasion in Latin America epitomize this region. The Urabá gulf witnessed the rise and death of Santa María de La Antígua (the oldest funded Spanish city of the Americas in *terra firma*, [Sarcina, 2017]), the spread of African‐descendant peoples that displaced indigenous groups after slavery abolition, the growth of coca cultivation and the linked problem of illegal armed groups and the rise of the region as a major trafficking illegal immigration corridor (Marciales, 2015; Uhm, 2020). In Sapzurro bay, these processes resulted in a complex mosaic composed of a small town inhabited by low‐income African‐descendant people, large scattered residences, tourist infrastructure, banana and coconut plantations and small patches of mangrove forests associated with small creeks. Although the bay as a hole could be best designated as ‘urban’ in character, it exhibits a range of land covers including both natural and built lands. Blue crabs are consistently observed in natural habitats, such as small mangrove patches predominantly featuring *Rhizophora mangle*, *Conocarpus erectus*, or *Avicennia germinans*. These crabs are also prevalent in cropland areas, including pastures, banana, and coconut plantations, as well as urban environments. In urban settings, they are often found in open sediment areas designated for various urban land uses (e.g., residential, recreational, industrial, commercial, and transportation), alongside impervious surfaces, sewage canals, and amidst houses. In proximity to residential areas, humans frequently provide food scraps to crabs and occasionally harvest them when they attain substantial size. Given these characteristics, areas inhabited by *C. guanhumi* where classified into three distinct land cover types, mangrove patches, croplands, and urban areas (see also Figure 1), for our subsequent analyses.

#### 2.1.2 Sampling strategy

To assess the differences in spatial distribution among land use types, data were collected in November 2019 at Sapzurro bay (Figure 1). We placed quadrats (area = 4 m^−2^) along 40 m transects placed at the supralitoral zone of the beach, where *C. guanhumi* is distributed. Transects and quadrats were placed in each type of land cover (mangrove patches: 5 transects with 20 contiguous quadrats, crop lands: 2 transects with 20 contiguous quadrats and urban lands: 2 transects with 20 contiguous quadrats. Unequal number of transects reflects the availability of crabs in each land use type). Owing to the status of *C. guanhumi* as threatened species and the restrictions to collect it, we used the number and diameter of burrows to estimate the relative abundance and body size – a widely used procedure validated by Govender and Rodríguez‐Fourquet (2008). Circular plastic plates with different diameters (30, 60, 100 mm) were used to rapidly classify and count the number of burrows within each quadrat in four size ranges: I up to 30 mm, II = >30–60 mm, III = >60–100 mm and IV = >100 mm. Finally, the total number of burrows in each quadrat was used to assess their spatial distribution pattern through the Local Quadrat Variance method that analyses changes in the variance of the total number of burrows when calculated through combinations of different quadrat block sizes (see below).

### 2.2 Literature compilation

The compilation was conducted following the recommendations of Khan et al. (2003) for systematic reviews. We established “*Cardisoma guanhumi*” and “blue crab” as primary keywords, combined with, “carapace width”, “density”, “abundance” and “density” as secondary keywords in our searches in the Google Scholar database. The search was conducted in English, Spanish and Portuguese language. Relevant literature, including scientific articles and unpublished documents (e.g., thesis, project reports, extended abstracts, proceedings) was identified by title and abstract, resulting in 38 documents of interest. Documents were included in the review and analyzed in more detail if they (i) contained estimations of abundance (individuals/burrows) per unit area, based on catches of crabs using traps or manual catches or tagging‐recapture experiments and/or they (ii) contained quantitative estimations of either average, maximum and minimum carapace width, based on direct measurements of crabs or on burrowing diameter. In three cases, where estimations were only presented in graphs, these were digitalized and then converted to numbers using the Digitizelt 2.5.9 program (Bormann, 2016). Studies not clearly reporting the methods used or the unit area/length being reported were excluded. Based on the description of the sampling area, each study site was classified into three types of land cover: natural habitats (including mangrove forests, grasslands, vegetated forests, swamps and ponds), croplands and urban areas (see Tables S1 and S2; Carmona‐Suárez, 2011; Carmona‐Suárez & Guerra‐Castro, 2018; Giménez et al., 2015; Hernández‐Maldonado, 2013; Zapata, 2018). In three registers (out of 111), the habitat description of the study site was lacking or not clear. Taking into account that this species has been most commonly studied in wild habitats, these sites were classified as natural habitats.

### 2.3 Data analysis

#### 2.3.1 Local scale differences among areas with different land covers

To test for differences in the relative abundance of *C. guanhumi* among types of land cover (mangrove patches, crop lands and urban lands) a Welch's ANOVA model was used because data did not meet the assumptions of parametric analyses of variance related to normal error distribution and homoscedasticity. When significant differences were found, we performed Games–Howell post‐hoc test, which is similar to Tukey's test, but does not assume equal variances and samples sizes. To analyze the differences in the size structure (the relative abundance of crabs in different size intervals) among land uses, the relative frequency of burrows in four size intervals in each land cover was estimated. Relative frequencies were used to account for the differences in the number of quadrats sampled in each land use. Thereafter, the frequencies of size ranges were compared among land uses using a log‐linear analysis, which allows the evaluation of frequency data in two‐factor cross‐tabulation table (4 × 3; i.e., four size intervals in three land use types).

To assess differences in spatial distribution among land use types the two‐term local quadrat variance analysis was used. This is a modification of the basic blocked quadrat variance method commonly used to describe spatial patterns in terrestrial ecological studies, which has a more refined blocking scheme in its calculation and has been used to assess spatial patterns of marine invertebrates (Butler et al., 1999). This analysis groups contiguous quadrats in each transect in increasingly larger groups of quadrats (patch size) and calculates a variance for each patch size. Plotting variance against patch size provides information on scale and pattern, with peaks in variance indicate clustering of quadrats at a given patch size.

#### 2.3.2 Large‐scale patterns of blue land crab populations

Kruskal–Wallis tests were used to assess the large‐scale differences in the mean abundance and body size among land uses. Moreover, tests for differences in mean abundance and body size among types of land cover where conducted at the country level to account for effects of ecological settings and latitudinal variability on these population parameters. Owing to the limited spatial replication for each land cover, tests where only conducted when enough data were available to enable a meaningful comparison. Mann–Whitney or Kruskal–Wallis tests, were used to compare mean abundance, minimum size, mean size and maximum size among the three or two types of land cover, respectively, sustaining crab populations in each country. Finally, simple regression analyses were used to assess if body size (mean, maximum and minimum size) changed significantly as a function of time (year). All statistical analyses were performed using program R (version 4.0.4), including the wilcox.test, rstatix, nortest, ggstatsplot, ggplot2, openxlxs and tidyverse packages (R Core Team, 2021) The R script implemented for the TTLQV analysis is provided as a File S1.

## 3 RESULTS

### 3.1 Local scale differences among areas with different land covers

Despite the strong modification of the shoreline by humans, large numbers of blue crabs were observed in natural habitats (small mangrove patches dominated either by *R. mangle*, *C. erectus* or *Avicenia germinans*) in croplands (pastures, banana and coconut crops) and urban areas (mainly in bare sediment surrounding impervious surfaces, along sewage canals and among houses).

**FIGURE 2:**
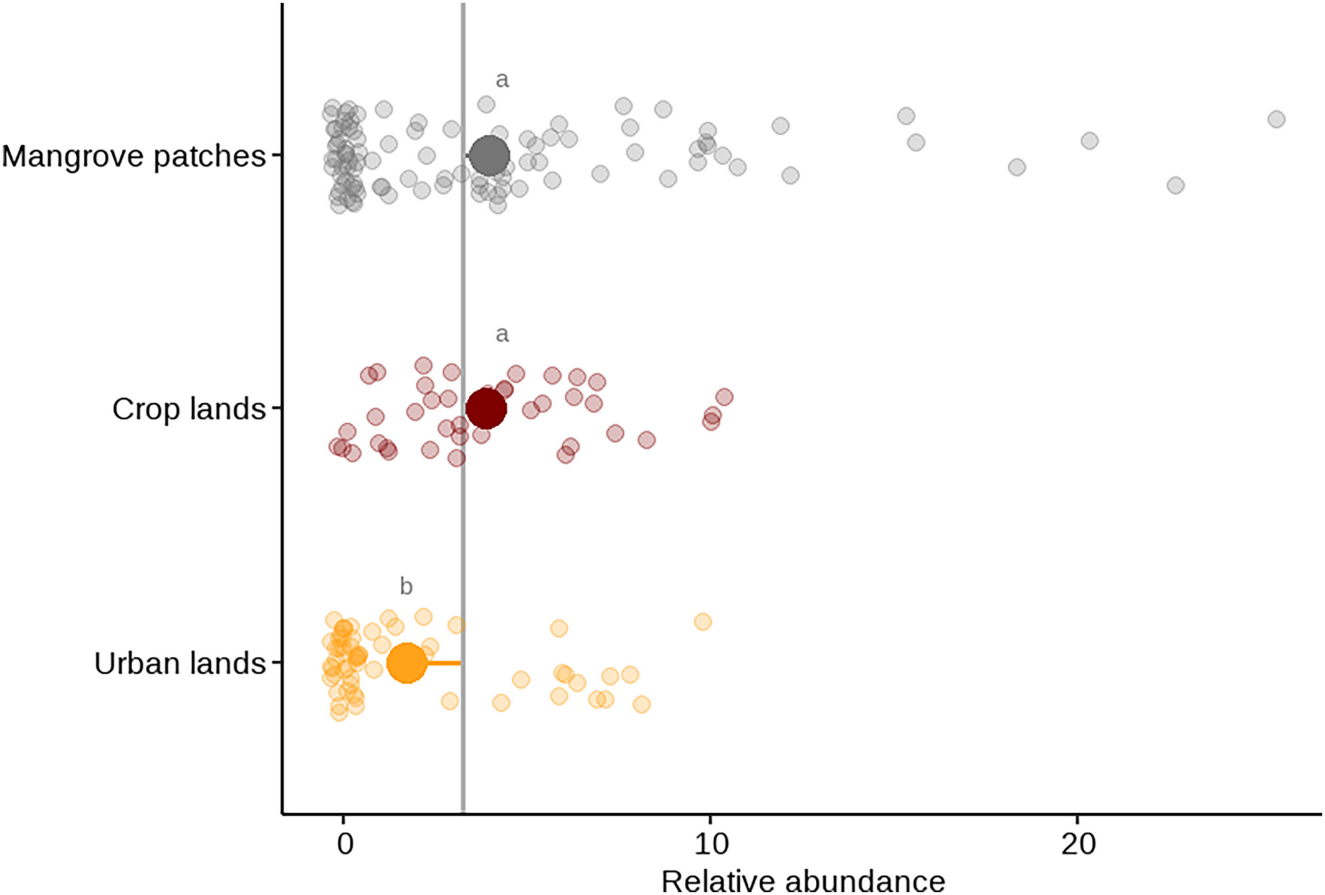
The relative abundance of *Cardisoma guanhumi* in areas with different land uses in Sapzurro bay. Abundances (estimated as the number of burrows per 4 m^2^ quadrant) are individual points for each land use. Gray line: overall mean; lollypops: mean for each type use and distance to the general mean. Land uses with the different letters are statistically different (*p* < .05) after Games–Howell post‐hoc test.

*C. guanhumi* showed significant differences in relative abundance among land uses (*F*_2,115_ = 9.53; *p* < .001). Urban lands showed a lower abundance of crab burrows (mean = 1.72 m^2^ [SD = 2.7]), compared to mangrove patches (3.94 m^2^ [5.29]) and crop lands (3.85 m^2^ [2.85]) (Figure 2). Noteworthy, there was more dispersion around the mean abundance for mangrove patches, with 6 quadrats (out of 100) showing a high abundance (>15 burrows m^−2^) and ~70 quadrats with a low abundance (<5 burrows m^−2^).

**FIGURE 3:**
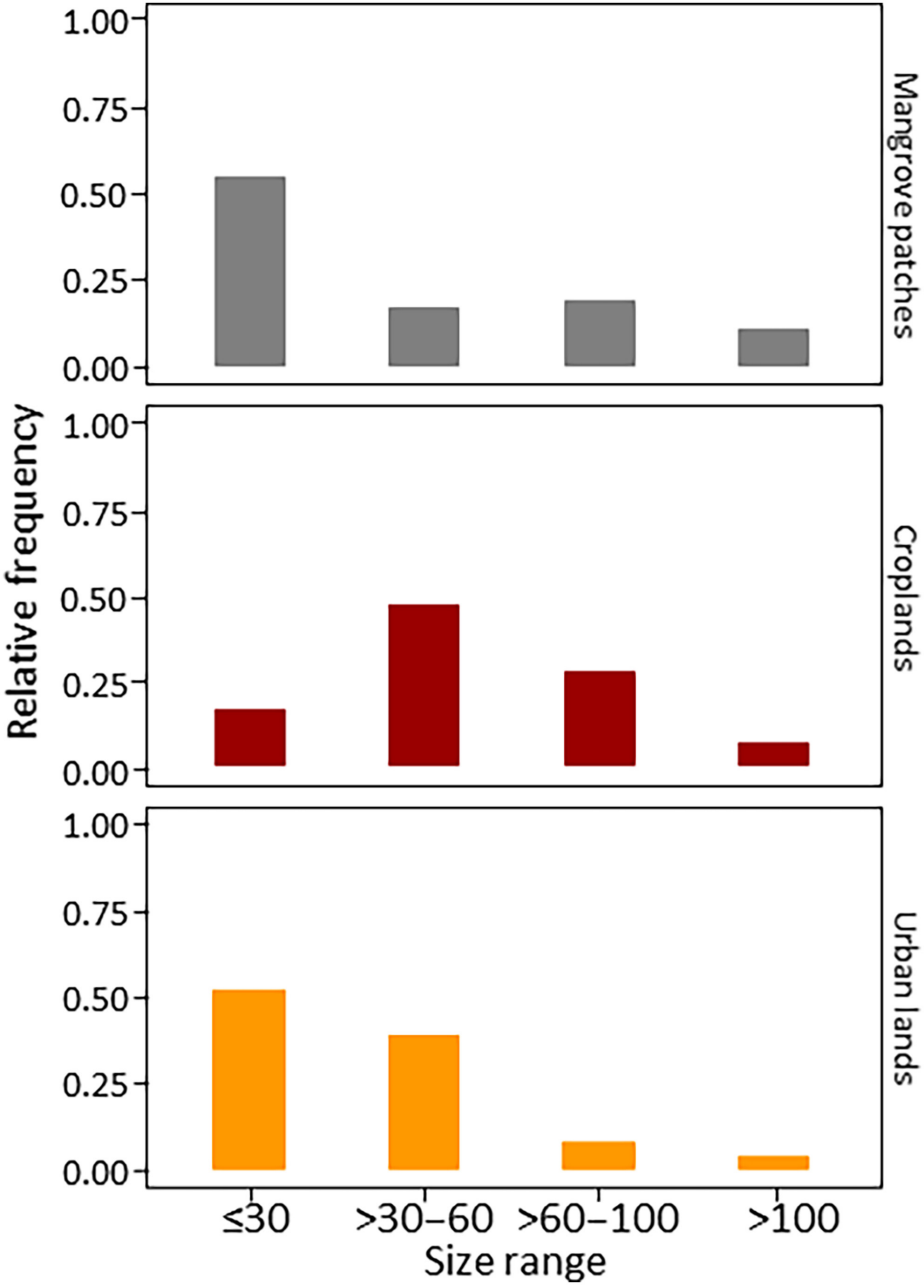
Comparison of the burrow size structure of *Cardisoma guanhumi* among land uses in Sapzurro bay. Bars represent the relative frequency of burrow's size ranges of crabs from places with three distinct land uses.

The size structure of burrows of *C. guanhumi* changed significantly among land uses (*χ* ^2^ = 191.07, df = 6, *p* < .001). Figure 3 shows that smaller burrows (Size I) were more frequent in urban lands and mangrove patches. In contrast, large burrows (Size III and IV) were more frequently observed in mangrove patches and crop lands.

**FIGURE 4:**
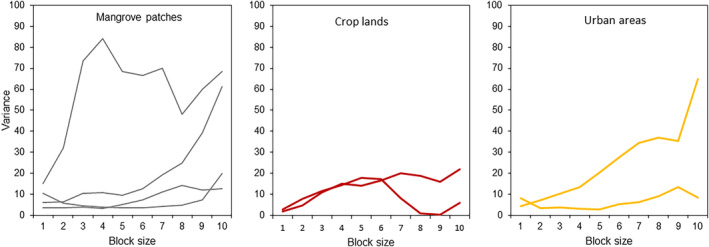
Comparison of the distribution patterns of *Cardisoma guanhumi* among land uses in Sapzurro bay. Variance‐block size plots for the distribution of burrows of crabs along transects located in areas with different land uses. Lines in each panel represent individual transects (40 m) located along the supralittoral zone.

The distribution of burrows of *C. guanhumi* in mangrove forests and urban lands generally showed patchiness along transects (Figure 4). However, there was high variability within each land use, particularly within mangrove forests, possibly hinting to the strong alteration of these habitats that results in many quadrants without burrows (see Figure 2). The variance in mangrove forests and urban lands generally increased and peaked toward the larger block sizes, implying that patch diameter is larger than 40 m. In contrast, the variance in crop lands showed little change, suggesting that the distribution of burrows was more evenly distributed, which is consistent with the fact that croplands were usually topographically modified and cultured to hold a single crop.

### 3.2 Large‐scale patterns of blue land crab populations

**FIGURE 5:**
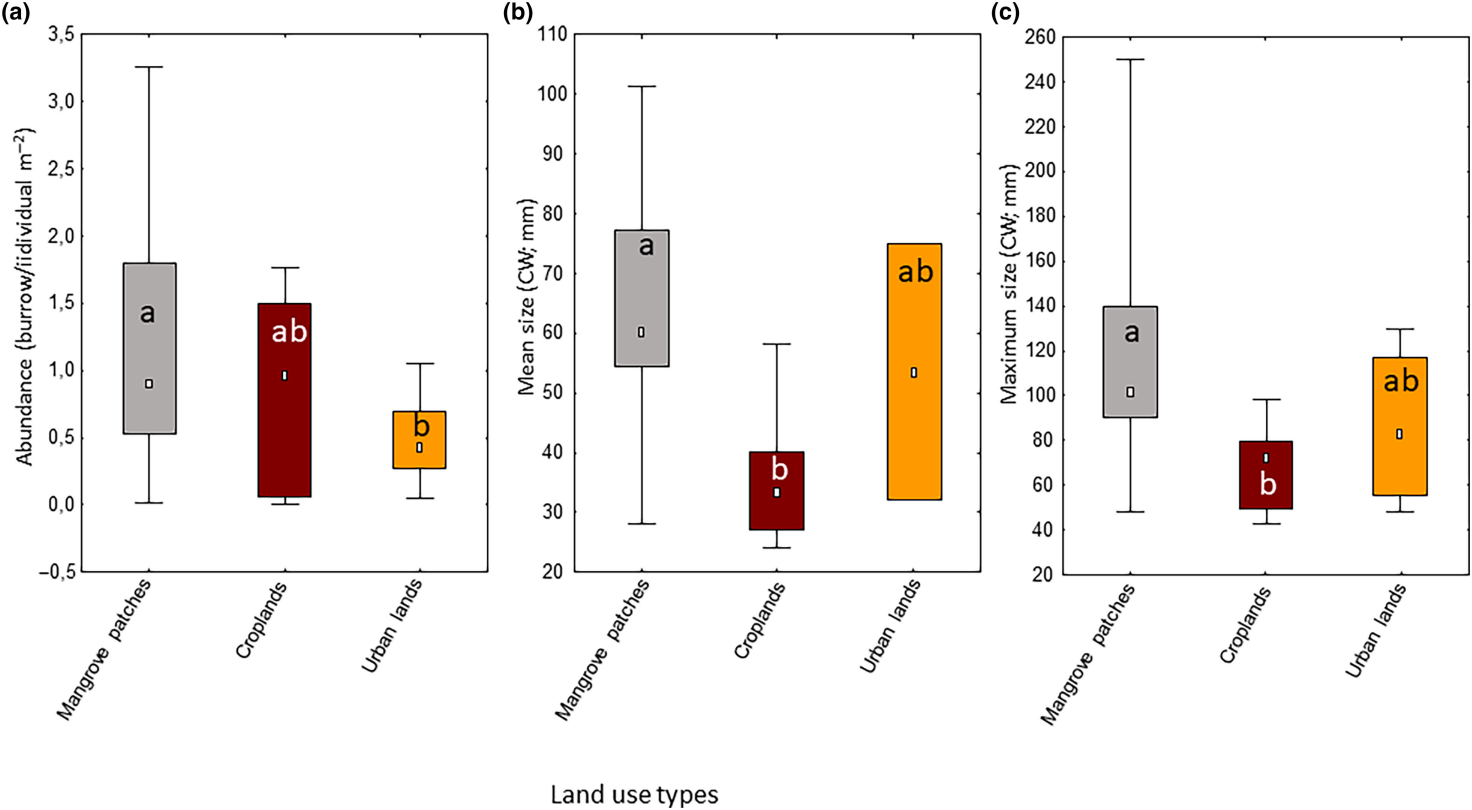
Large‐scale differences in populations of *Cardisoma guanhumi* among land use types. (a) Differences in the abundance of crabs among land uses. (b) Differences in mean size of carapace width (CW; mm) among land uses. (c) Differences in maximum size of carapace width (CW; mm) among land uses. Different letters within box plots show significant differences between levels of each variable, after post‐hoc pared comparisons of mean ranks. White dots within boxes represents the median, boxes represent the 25–75 percentiles and whiskers represent the minimum and maximum values. Data used for this analysis are given in Tables S1 and S2.

Our systematic review rendered 25 studies suitable for further analysis, composed of 18 scientific articles (13 in English, 3 in Spanish and 2 in Portuguese) and 7 unpublished documents in Spanish (2 MSc thesis, 4 BSc thesis, 1 conference proceeding). These studies provided information on abundance and size ranges of *C. guanhumi* for the whole geographic distribution range in the Americas for the last 73 years. At large‐scale, we found significant differences in crabs' abundance and body size among land uses (Table 1). Mean abundance was lower in urban lands (mean = 0.478 [SD = 0.295]), compared with mangrove patches (1.165 [0.842]), while mean and maximum size were lower in croplands (36.37 mm [11.233] and 67.42 mm [19.18]) than those in mangrove patches (63.20 mm [17.13] and 115.18 mm [43.5]) (Figure 5).

**TABLE 1:** Results of Kruskal–Wallis ANOVA by ranks for large‐scale comparisons of the mean abundance and body size of *Cardisoma guanhumi* among land cover types from literature review (see Tables S1 and S2).

Parameter	Mean values (standard deviation)			*N*	*H*	*p*‐Value
	Mangroves	Croplands	Urban lands			
Abundance	1.16 (0.83)	0.81 (0.73)	0.48 (0.29)	54	6.988	**.030**
Mean size	63.19 (17.13)	36.36 (11.23)	53.50 (30.40)	50	10.326	**.006**
Maximum size	115.17 (43.51)	67.42 (19.18)	89.00 (57.98)	57	11.548	**.003**

*Note*: Tests showing significant differences are reported in boldface.

**FIGURE 6:**
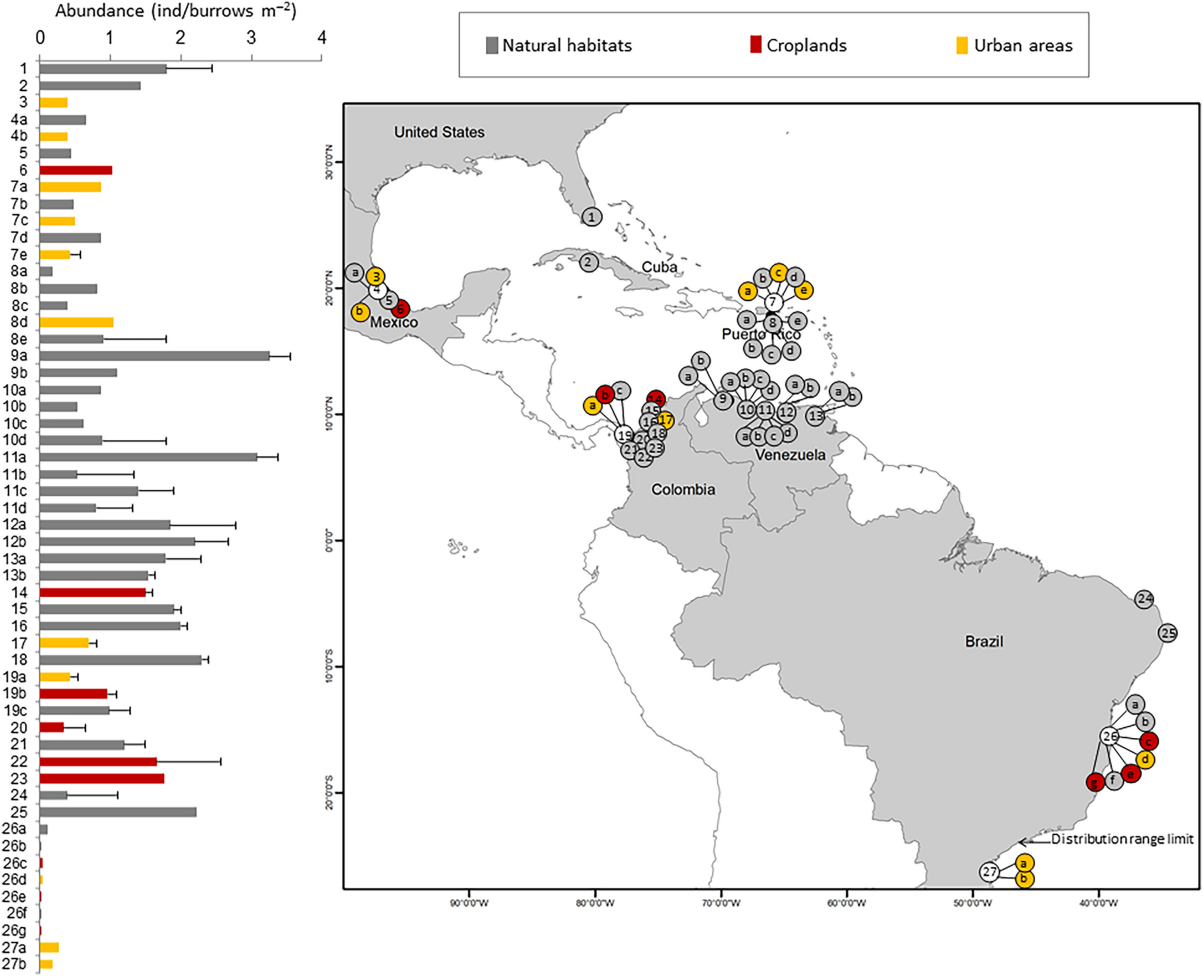
Large‐scale north–south variability in the abundance of Cardisoma guanhumi among land uses. Left panel: Bars represent the mean abundances of crabs inhabiting natural habitats (gray bars), croplands (red bars), and urban areas (yellow bars) in different locations throughout the species' distribution range. Error bars correspond to standard deviations; bars without error lack measures of variability around the mean in the original study (Table S1; Barrios, 2008; Morales‐Costa & Schwamborn, 2018). Codes on the vertical axis correspond to study locations displayed as bubbles in the right panel. Different numbers denote different study locations, and letters denote distinct study periods or land uses for the same location. The color keys for bubbles (gray, red, yellow) follow the same pattern as the bars in the left panel, with white color denoting mixed land uses in a given location. Codes for locations are as follows: 1: Dade County, Florida (USA); 2: Bahía Cochinos (Cuba); 3: Barra de Galindo, Veracruz, (Mexico); 4: Tampamachoco Lagoon, Veracruz (Mexico); 5: Tumilco, Veracruz (Mexico); 6: Majahual, Veracruz (Mexico); 7: San Juan Bay (Puerto Rico); 8: Jobos Bay (Puerto Rico); 9: West Coast (Venezuela); 10: Triste Gulf (Venezuela); 11: Miranda (Venezuela); 12: Central Coast (Venezuela); 13: East Coast (Venezuela); 14: Agrotijo, Cispatá (Colombia); 15: Caño Soldado, Cispatá (Colombia); 16: Caño Tijó, Cispatá (Colombia); 17: CVS, Cispatá (Colombia); 18: Helechal, Cispatá (Colombia); 19: Sapzurro, Chocó (Colombia); 20: Camerún, Turbo (Colombia); 21: Punta de Piedra, Turbo (Colombia); 22: Cirilo, Turbo (Colombia); 23: Tie, Turbo (Colombia); 24: Imburana, Rio Grande do Sul (Brazil); 25: Itamaracá, Pernambuco (Brazil); 26: Southern Bahía (Brazil); 27: Babitonga Bay, Santa Catarina (Brazil). Bibliographic references for each study site are provided in Table S1.

In a latitudinal context, the abundance was higher in Venezuela, Colombia, and northern Brazil and decreased toward the geographic range limits, particularly the southern limit (Figure 6). However, there were no significant differences in the abundance of *C. guanhumi* among land uses in any of the evaluated countries (Table 2). Notably, urban crab populations from Babitonga bay (Brazil), which represent a range extension, were much more abundant (mean = 0.220 burrows m^−2^) than those from natural habitats (mean = 0.053 burrows m^−2^) and croplands (mean = 0.021 burrows m^−2^) in Bahía, located nearly 2000 km north of São Paulo (the southern distribution range limit).

**TABLE 2:** Within‐country comparisons of the mean abundance and body size of *Cardisoma guanhumi* among types of land cover.

Parameter	Country	Kruskal–Wallis test	Mann–Whitney *U* test
Mean abundance	Colombia	*H*(2) = 4.70; *p* = .10	
Mean abundance	Brazil	*H*(2) = 3.40; *p* = .18	
Mean abundance	Puerto Rico		*U* = 8.50; *p* = .50
Maximum body size	Brazil	*H*(2) = 3.55; *p* = .17	
Maximum body size	Colombia		*U* = 0.00; ***p* = .04**
Mean body size	Brazil	*H*(2) = 2.22; *p* = .33	
Mean body size	Colombia		*U* = 2.00; *p* = .14
Minimum body size	Brazil	*H*(2) = 3.13; *p* = .21	
Minimum body size	Colombia		*U* = 1.00; *p* = .07

*Note*: Tests showing that significant differences are reported in boldface.

**FIGURE 7:**
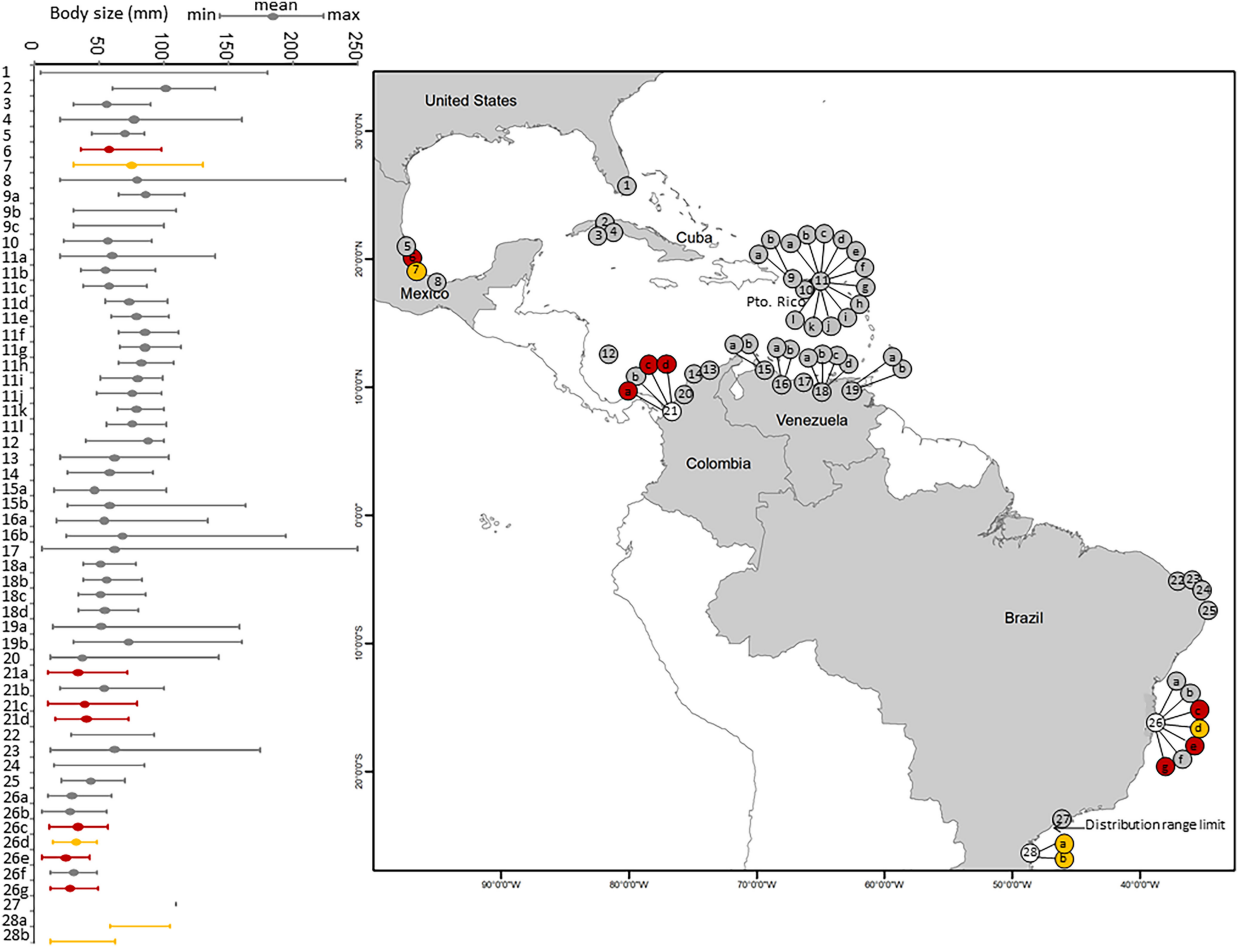
Large‐scale north–south variability in body size parameters of *Cardisoma guanhumi* in different land uses. Mean size (oval), minimum size (left whisker), and maximum size (right whisker) correspond to the carapace width (mm) of crabs inhabiting natural habitats (gray), croplands (red), and urban areas (yellow) in various locations throughout the species' distribution range. Ovals/whiskers are omitted when the corresponding estimation was not available in a given document. The southern distribution range limit given corresponds to São Paulo (Rathbun, 1918). Codes for locations are as follows: 1: Dade County, Florida (U.S.A); 2: El Maíz, Zapata (Cuba); 3: Guamutal, Zapata (Cuba); 4: La Arenera, Zapata (Cuba); 5: Tampamachoco, Veracruz, (Mexico); 6: Majahual, Veracruz (Mexico); 7: Tuxpan, Veracruz (Mexico); 8: Sontecomapan, Veracruz (Mexico); 9: Puerto Rico; 10: Maunabo (Puerto Rico); 11: Jobos Bay (Puerto Rico); 12: San Andrés Island (Colombia); 13–14: Tayrona‐Salamanca National Parks, Magdalena (Colombia); 15: West Coast (Venezuela); 16: Central Coast (Venezuela); 17: Triste Gulf (Venezuela); 18: Miranda (Venezuela); 19: East Coast (Venezuela); 20: Cispatá Bay (Colombia); 21: Turbo, Antioquia (Colombia); 22: Cumbe, Ceara, (Brazil); 23: Imburana, Rio Grande do Sul (Brazil); 24: Natal, Rio Grande do Sul (Brazil); 25: Itamaracá, Pernambuco (Brazil); 26: Southern Bahía (Brazil); 27: São Paulo (Brazil); 28: Babitonga Bay, Santa Catarina (Brazil). Bibliographic references for each study site are provided in Table S2; Cardona et al., 2019; Hernández‐Maldonado & Campos, 2015; Morales‐Costa & Schwamborn, 2016; Quiñones‐Llópiz & Rodríguez‐Fourquet, 2019; Salinas, 2012; Shinozaki‐Mendes et al., 2013; Silva et al., 2014.

Body size showed a high variability through the distribution range of *C. guanhumi* (Figure 7), with shorter size ranges observed in island territories (Cuba and Puerto Rico) and larger sizes mostly observed in regions from lower latitudes. At the country level, there were generally no significant differences in the body size parameters of *C. guanhumi* among land uses in Brazil and Puerto Rico, except for the maximum body size in Colombia (Table 2), where natural habitats displayed higher maximum sizes (107.63 mm carapace width) than croplands (91.72 mm carapace width). Again, the maximum body size observed in urban populations from Babitonga bay (mean = 83.50 mm) were larger than those located north of the southern distribution range limit in natural habitats (mean = 54.83 mm) or croplands (mean = 49.66 mm).

**FIGURE 8:**
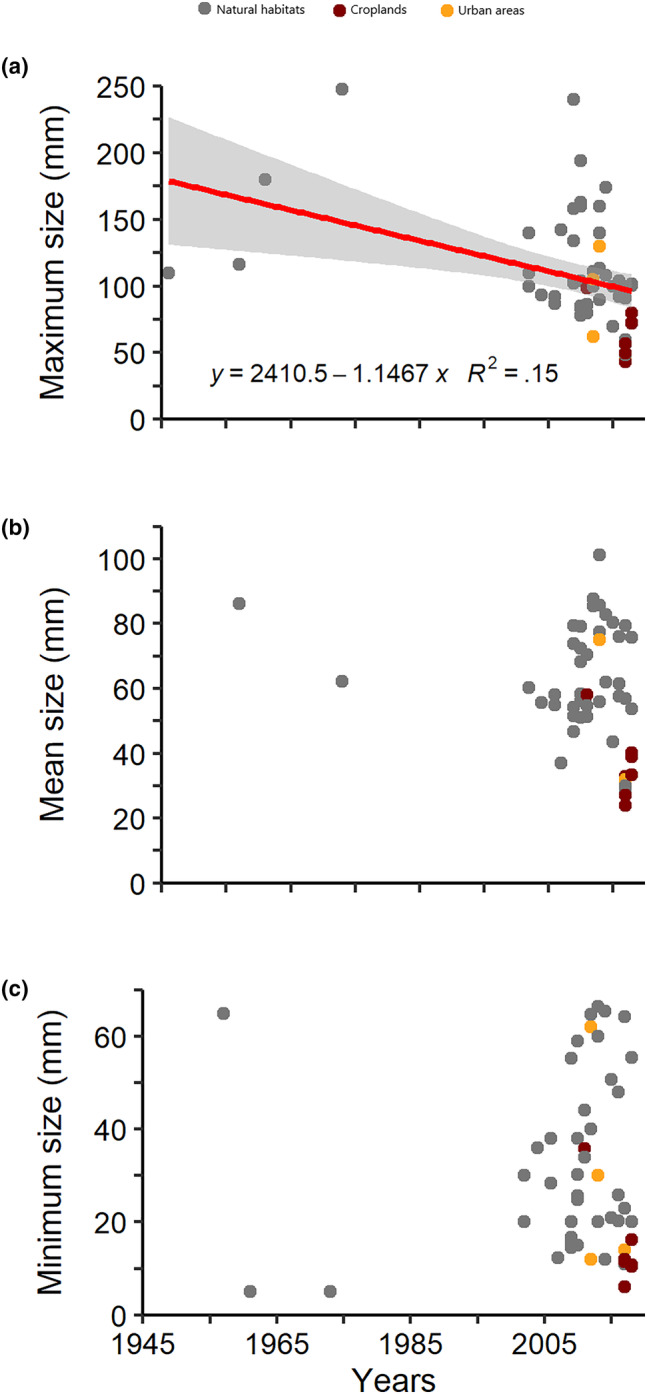
Changes in maximum (a), mean (b) and minimum size (c) of *Cardisoma guanhumi* through time. Dot color represent estimations performed by individual studies in mangrove patches (gray), croplands (red) and urban areas (yellow) through its geographic distribution range. Significant relationships show the regression line with the associated statistics and 95% confidence interval. Data points represent individual studies gathered in Table S2.

Figure 8 shows the variability of body size as a function of time. Although there are different limitations and sources of variability involved in such an analysis (i.e. data are unevenly distributed through years, different methods were used for estimations, high spatial variability expected), our results indicate that maximum body size of *C. guanhumi* decreased significantly during the last 73 years (*F*_1,55_ = 9.525; *p* = .003). The significance in the reduction of maximum body size through time may be thought as dependent on the few data available between 1940 and 1980, but an analysis restricted to data after 2000, when most data were taken, showed a more robust relationship (*F*_1,51_ = 9.634; *p* = .003). Moreover, a significant negative relationship was still obtained after excluding estimations from urban habitats and croplands (*F*_1,44_ = 5.353; *p* = .025). In contrast, the average (*F*_1,48_ = 3.653; *p* = .061) and minimum size (*F*_1,54_ = 0.016; *p* = .9) did not change significantly with time.

## 4 DISCUSSION

Despite the centennial, substantial human transformation of the coastline in Sapzurro bay and its status as vulnerable species in Colombia (Ardila et al., 2002), *C. guanhumi* seems to be successfully thriving in areas with local‐scale differences in land cover. The ability of *C. guanhumi* to occupy diverse inland habitats, provided that they have access to the groundwater table, has been widely reported (Govender et al., 2008; Herreid & Gifford, 1963; Mendez & Cruz, 2017; Novais et al., 2021; Rodríguez‐Fourquet & Sabat, 2009).

In our study, the occupation of habitats devoid of vegetation among human residences in highly modified urban lands is remarkable for a number of reasons. First, the higher frequency of smallest burrows in urban lands suggest that these are suitable recruitment grounds, a critical factor for population stability at local scales (Yau et al., 2014). Second, the lower abundance and lower frequency of largest burrows in urban areas as compared with croplands and mangrove patches and the observation of people feeding crabs suggest a process of livestock farming. This seems a long‐term process rooted in cultural patterns of African‐descendent peoples in South America: Oliveira (1946) chronicled that locals of the suburbs of Sao Paulo (Brazil) used to build barrels surrounded by bamboo and canvas to maintain crabs that are fed with food remains and similar findings were reported by Feliciano (1962) in Puerto Rico and Taissoun (1974) in Venezuela. Third, patchiness has been proposed to reflect physical factors (shading, distance to rivers or other water bodies, substrate properties facilitating burrowing) or the distribution of native vegetation serving as food (Novais et al., 2021; Oliveira‐Neto et al., 2014). However, we found patchiness in urban areas, which present highly modified biophysical factors and novel materials, as compared to natural habitats (Alberti, 2008, Blanco‐Libreros et al., 2021; Riascos & Gomez, 2023). The existence of shelters, e.g. below concrete structures, seems a factor contributing to a gregarious behavior in urban habitats, but our results on small‐scale spatial distribution must be taken cautiously, as patterns of spatial distributions may exhibit dynamics that were not captured by short‐term samplings.

Our study suggests that urban habitats – without vegetation that may mimic forests and associated resources/conditions in rural areas – are providing opportunities for the persistence of threatened species at local scales; the behavioral shift to use novel food sources, seems a major determinant of these proliferations in urbanized systems, as observed by Riascos and Gomez (2023).

The higher abundance of *C. guanhumi* observed in Venezuela, Colombia and northern Brazil as compared with higher latitudes in the northern and southern hemispheres generally conforms to the abundant‐centre hypothesis, which states that species perform better at the centre of their range, where high abundances are observed, and that these decline toward the range edges (Brown, 1984). However, the northern and southern range edges of this coastal land crab display contrasting features. The exploitation pressure in United States is low because the crab is either considered a pest or its fishery is strongly regulated (Hostetler et al., 2003), while exploitation is strong in Brazil, where fisheries are intensive and unregulated (Firmo et al., 2012). Moreover, many coastal habitats in the northern range edge are insular, with limited river discharge, whereas those in the southern range edge occur in mainland shorelines receiving substantial sediment and nutrient loads. In any case, several studies have questioned the empirical support and generality of the demographic predictions derived from the abundant‐centre hypothesis (Santini et al., 2019), and therefore explaining the latitudinal differences among populations fall outside the scope of our work. At a large scale, our findings indicate a lower abundance of crabs and smaller body sizes in urban and cropland areas, respectively. However, it's important to take these results with caution. Most studies reporting these parameters for urban and cropland areas are mostly located close to the northern or southern species distribution ranges (see Figures 6 and 7), where crabs tend to be less abundant and exhibit smaller body sizes for all land use types. Consequently, whether variations in crab abundance and body size exist among different types of land use remains an open question in our view.

In contrast to the dominant discourse of habitat transformation as a causative factor for population decline our results showed no differences in the abundance of *C. guanhumi* among land uses at the country level, thus suggesting that croplands and urban areas are suitable habitats for this threatened species across different spatial scales. In fact, our results suggest that urban habitats provide the conditions for a range expansion in the southern distribution range limit. This is not surprising; there is increasing evidence that tropical species, including coastal crabs, are generally expanding their distribution ranges to higher latitudes under on‐going warming trends in ocean conditions (e.g., Cheung et al., 2009; Osland et al., 2021; Riley et al., 2014; Schoeman et al., 2015; Tewksbury et al., 2008). It is surprising that human‐disturbed systems seem to offer habitats and resources that increase the fitness of edge populations and hence act as stepping stones for geographic range extensions, as observed for populations of the mangrove tree crab (*Aratus pisonii*) inhabiting docks in salt marsh ecosystems (Cannizzo et al., 2020). We argue that among the diverse array of local alterations related to urbanization, the long‐term provision of novel food sources is driving changes in demographic processes of *C. guanhumi* across scales. First, the large amount of materials and energy that cities metabolize begets a persistent supply of organic wastes (Alberti, 2008), including food remains, dead animals and human feces that can be eaten by these crabs (Firmo et al., 2012; Oliveira, 1946 and see Figure S1). Second, local populations persisting in polluted urban habitats are commonly rejected by consumers (Aquino, 2015; Firmo et al., 2012). Thus, the adaptive plasticity allowing habitat and dietary shifts result in a halt in exploitation while maintaining the food provision from humans, with obvious consequences for crab's abundance and size structure. Third, while urbanization is a contemporary process, vestiges of the association of this crab with humans are traced back to the Teotihuacan Mesoamerican city (Rodríguez‐Galicia et al., 2017) and Pleistocene cave deposits in Puerto Rico (Schweitzer et al., 2008), which suggest that the exploitation of this species and perhaps also the species responses to human‐related disturbances may have a fairly long history.

While spatial comparisons suggest that crab populations are thriving in heavily disturbed habitats, the temporal analysis suggest an endured reduction of maximum body size as a function of time. It is worth noting that a decrease in crab size since the 1950's has been reported at a local scale (Govender, 2019). Our finding is in line with a widely observed reduction of body size in wild animals as a response to different types of human pressures. The first, recognized as an outcome of human‐induced evolution induced by unnatural selection through harvesting of larger animals (e.g. Allendorf & Hard, 2009). The second, representing the phenotypic signature of animals thriving urban habitats where environmental settings, particularly thermal regimes, are shifted (e.g., Alberti et al., 2020; Ramos et al., 2021). The latter would explain why the slope of the size‐time relationship is steeper when estimations from urban and croplands are included.

## 5 CONCLUDING REMARKS

In the big picture, our results add to mounting evidence that threatened species are either behaving as urban‐dwelling animals or developing local urban populations (Busschots et al., 2020; Geary et al., 2021; Ives et al., 2016; Maclagan et al., 2018; Planchuelo et al., 2019; Van Helden et al., 2020). This trend is not new (e.g., Luniak, 1996; Witt, 1991), but it has been largely neglected in conservation biology, epitomizing a long intellectual tradition in conservation biology of focusing on wilderness, wild species in natural ecosystems – a philosophical approach termed by Callicott et al. (2000) as compositionalism, in which human impact fall outside the sphere of interest or even ignite rejection to biodiversity thriving in human‐dominated systems (Riascos et al., 2020). Recognizing that the future of some threatened species may be defined beyond the boundaries of parks and reserves is an uncomfortable truth that emphasized the long‐established need to address conservation in human‐dominated systems (see Robinson, 2006).

Recognizing that nearly 40% of potential terrestrial net primary productivity is used to sustain human activities (Krausmann et al., 2013; Vitousek et al., 1986) and that most of this flux of energy and materials is mostly metabolized in large urban systems (Imhoff et al., 2004) may help us to understand the adjustment of wild animal populations to specific conditions of urban environment. Cities represent a fundamentally new, expanding and relatively vacant habitat characterized by high anthropogenic pressure, but also by a diverse and abundant flux of food that must be attractive for wild species. Shifts in feeding strategies leading to the exploitation of anthropogenic food is being recognized as a major driver of urbanization of wild species (Carmona et al., 2021; Stofberg et al., 2019). As urbanization, at the scale of landscape, has occurred only during the last ~200 years and urban expansion is accelerating we argue that urban biotic assemblages will be diversifying over time – a hypothesis advanced by Luniak (1996, 2004). Perhaps the best illustration of the strength and global character of this cryptic trend aroused during the “anthropause” (sensu Rutz et al., 2020): the dramatic global sowing of modern human activity during COVID‐19 lockdowns made visible how an unsuspected diversity of animals are able to use or are reliant on anthropogenic food sources (Silva‐Rodríguez et al., 2021; Soh et al., 2021).

## AUTHOR CONTRIBUTIONS

**José M. Riascos:** Conceptualization (lead); data curation (lead); formal analysis (lead); investigation (lead); methodology (equal); writing – original draft (lead); writing – review and editing (lead). **Levy D. Obonaga:** Conceptualization (supporting); data curation (supporting); formal analysis (supporting); investigation (supporting); methodology (supporting); writing – original draft (supporting); writing – review and editing (supporting). **Jhostin Ramos:** Conceptualization (supporting); data curation (supporting); formal analysis (supporting); investigation (supporting); methodology (supporting); writing – original draft (supporting); writing – review and editing (supporting).

## CONFLICT OF INTEREST STATEMENT

The authors declare that the research was conducted in the absence of any commercial or financial relationships that could be construed as a potential conflict of interest.

## Supporting information


Figure S1.

File S1.

Table S1.

Table S2.

## Data Availability

All the raw data supporting this work are provided as [Link], [Link], [Link], [Link].
