# Supplementary material for: Is the threatened land crab Cardisoma guanhumi conquering human‐dominated systems?
Source: Ecol Evol. 2024 Apr 25;14(4):e10737. doi: 10.1002/ece3.10737 (PMC11046080; doi:10.1002/ece3.10737)
Supplement: Supplementary file 1 — Figure S1. [file ECE3-14-e10737-s004.docx]

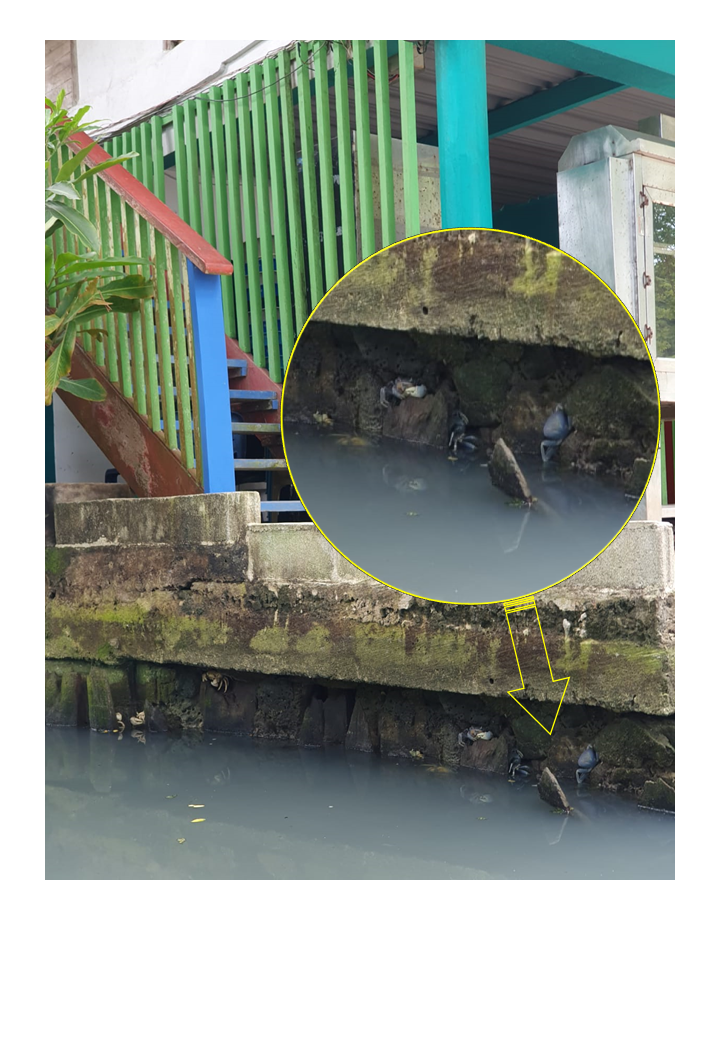


Figure S1. *Cardisoma guanhumi* inhabiting urban areas in Sapzurro, Chocó -Colombia. In this picture, crabs built their burrows among stones along a sewage channel. Picture: Charlotte Hopfe, August 2021
